# Complete chloroplast genome sequence of *Procris crenata* C.B.Rob (Urticaceae)

**DOI:** 10.1080/23802359.2020.1871436

**Published:** 2021-02-09

**Authors:** Long-Fei Fu, Zhi-Xiang Zhang

**Affiliations:** a Laboratory of Systematic Evolution and Biogeography of Woody Plants, College of Nature Conservation, Beijing Forestry University, Beijing, China; b Guangxi Key Laboratory of Plant Conservation and Restoration Ecology in Karst Terrain, Guangxi Institute of Botany, Guangxi Zhuang Autonomous Region and Chinese Academy of Sciences, Guilin, China

**Keywords:** plastid genome, Elatostema s.l, phylogeny

## Abstract

The complete chloroplast (cp) genome of *Procris crenata* C.B.Rob was reported. The cp genome was 154,124 bp in length and contained two inverted repeats (IRs) of 25,626 bp, which were separated by large single-copy and small single-copy of 84,599 bp and 18,273 bp, respectively. The GC content was 36.5%. A total of 113 functional genes were encoded, comprising 79 protein-coding genes, 30 tRNA genes, and four rRNA genes. This is the first reported plastid genome in *Procris* (Urticaceae), which will be useful data for resolving the relationship within the family.

*Procris* Comm. ex Juss. (Urticaceae) is a small genus with about 20 species of subshrubs, shrubs, epiphytic, and epilithic (Chen et al. 2003). It is distributed throughout warm-temperate and tropical regions of the Old World (Chen et al. 2003). As the member of tribe Elatostemteae, *Procris* has long been controversial with respect to *Elatostema* J.R.Forst. & G.Forst., *Elatostematoides* C.B.Rob., and *Pellionia* Gaudich (Hadiah et al. 2003, 2008; Hadiah and Conn 2009; Wu et al. 2013). Using three DNA regions, Tseng et al. (2019) conducted an extensive phylogenetic study demonstrating that *Procris* is a monophyletic genus and also clarifying the genus level of *Procris*, *Elatostema*, and *Elatostematoides*. However, the phylogenetic relationships within *Procirs* still remained poorly resolved which hampered our understanding on the evolution of the genus and the family.

Plastome provides sufficient information to resolve the phylogenetic relationships at generic and infrageneric level. Despite several plastid genomes of species in Urticaceae have been reported (Wu et al. 2017; Wu et al. 2018; Fu et al. 2019; Wang et al. 2020), none of them belong to *Procris*.

In this study, leaves of *Procris crenata* C.B.Rob were collected from Longzhou County, Guangxi, China (N106°46′1″, E22°44′24″). A specimen was deposited at the herbarium of Guangxi Institute of Botany (IBK, http://www.gxib.cn/spIBK/, contact person and email: Long-Fei Fu, longfeifu@126.com) under the voucher number XZB20180127-10. Genomic DNA was extracted using the modified CTAB method (Chen et al. 2014) and sent to Majorbio Company (http://www.majorbio.com/, China) for next-generation sequencing. Short-insert (350 bp) paired-end read libraries preparation and 2 × 150 bp sequencing were performed on an Illumina (HiSeq4000) genome analyzer platform. Raw data (approximately 2 Gb) was filtered using the FASTX-Toolkit to obtain high-quality clean data (http://hannonlab.cshl.edu/fastx_toolkit/download.html). The original data (SRR12846062) were mapped to plastid genome reference (*Elatostema dissectum*, GenBank-MF227819) in Geneious Primer (Kearse et al. 2012) to exclude nuclear and mitochondrial reads. Putative plastid reads were then used for *de novo* assembling construction. Contigs were able to be concatenated by the overlaps using the Repeat Finder program implemented in Geneious Primer until a ∼130kb contig (including SSC, IR and LSC) being built. The IR region was determined by the Repeat Finder program in Geneious Primer and was reverse copied to obtain the complete chloroplast genome. The annotation approach was performed using CPGAVAS2 and PGA (Qu et al. 2019; Shi et al. 2019).

The complete chloroplast genome of *Procris crenata* was 154,124 bp in length (GenBank-MW114890), the GC content was 36.5%. Large single-copy and small single-copy were 84,599 bp and 18,273 bp respectively, while IR was 25,626 bp in length. The plastid genome encoded 113 functional genes, including 79 protein-coding genes, 30 tRNA genes, and four rRNA genes.

The maximum likelihood phylogeny was reconstructed by the dataset of 80 encoded protein genes from 21 species of Urticaceae and 26 species of other families in Rosales (Figure 1). The result was congruent with previous studies showing that *Procris* was closely related to *Elatostema*. And they together formed a clade representing tribe Elatostemateae which was sister to tribe Urticeae (Wu et al. 2013; Tseng et al. 2019). The newly reported plastid genome will provide additional data for further study on the phylogeny and evolution of the genus and the family.

**Figure 1.**
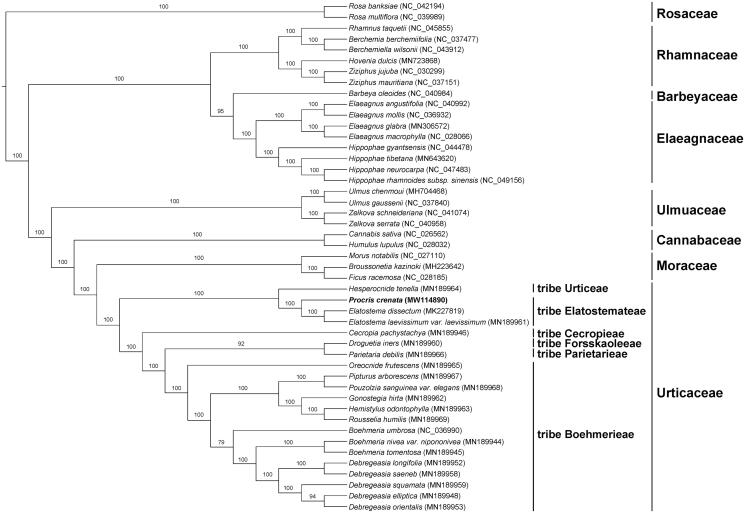
Phylogenetic tree reconstructed by maximum likelihood (ML) analysis based on the dataset of whole-chloroplast protein-coding genes from 21 species of Urticaceae and 26 species of other families in Rosales, numbers upon branches are assessed by ML bootstrap.

## Data Availability

The genome sequence data that support the findings of this study are openly available in GenBank of NCBI at (https://www.ncbi.nlm.nih.gov/) under the accession no. MW114890. The associated BioProject, SRA, and Bio-Sample numbers are PRJNA669252, SRS7538264, and SAMN16443195 respectively.
